# Tumor therapeutic response monitored by telemetric temperature sensing, a preclinical study on immunotherapy and chemotherapy

**DOI:** 10.1038/s41598-023-34919-w

**Published:** 2023-05-12

**Authors:** Qi Shao, Mia Lundgren, Justin Lynch, Minhan Jiang, Mikael Mir, John Bischof, Michael Nelson

**Affiliations:** 1 Department of Mechanical Engineering, University of Minnesota, Minneapolis, USA; 2 Department of Radiology, University of Minnesota, Minneapolis, USA; 3 School of Medicine, University of Minnesota, Minneapolis, USA; 4 Department of Biomedical Engineering, University of Minnesota, Minneapolis, USA

**Keywords:** Biomedical engineering, Immunotherapy, Preclinical research, Biological physics, Predictive markers, Prognostic markers, Immunosurveillance

## Abstract

Temperature in the body and the tumor reflects physiological and pathological conditions. A reliable, contactless, and simplistic measurement system can be used for long-term monitoring of disease progression and therapy response. In this study, miniaturized battery-free wireless chips implanted into growing tumors on small animals were used to capture both basal and tumor temperature dynamics. Three preclinical models: melanoma (B16), breast cancer (4T1), and colon cancer (MC-38), were treated with adoptive T cell transfer, AC-T chemotherapy, and anti-PD-1 immunotherapy respectively. Each model presents a distinctive pattern of temperature history dependent on the tumor characteristic and influenced by the administered therapy. Certain features are associated with positive therapeutic response, for instance the transient reduction of body and tumor temperature following adaptive T cell transfer, the elevation of tumor temperature following chemotherapy, and a steady decline of body temperature following anti-PD-1 therapy. Tracking in vivo thermal activity by cost-effective telemetric sensing has the potential of offering earlier treatment assessment to patients without requiring complex imaging or lab testing. Multi-parametric on-demand monitoring of tumor microenvironment by permanent implants and its integration into health information systems could further advance cancer management and reduce patient burden.

## Introduction

Temperature changes in the body have been recognized as an indicator of many diseases for centuries^1^. For cancerous tumors, localized temperature variations, both within the tumor and the body, are common due to changes in blood perfusion and metabolic heat generation. Cancer therapy can cause changes in energy balance dynamics within the tissue, resulting in a therapeutic response that can be monitored by tracking the temperatures^2^.

We hypothesize that tumor temperature change could be an early predictor of therapeutic response, such as immunotherapy, before the changes in size can be tracked by various imaging techniques. However, despite the wide range of available temperature measurement techniques that exist, monitoring tumor treatment response reliably by measuring temperature is uncommon. Approaches are ineffective in monitoring the tumor temperature through its progression and treatment.

Physiological temperatures are measured via either a contact-based or contactless manner. Contact-based approaches (e.g., conventional thermometer, thermistor, or thermocouples) are either invasive or can only measure surface^3^. Non-contact approaches, such as thermography used in breast cancer screening, can only measure the skin temperature but cannot reliably measure the internal region of the tumor. Other imaging-based approaches, such as MR thermometry^4^ and photoacoustic thermometry^5^, are either too expensive or too complicated to operate, making them not suited for monitoring deep body temperature over the time course of days to weeks. Therefore, a simplistic measurement system capable of providing real-time direct internal temperature is needed for long-term monitoring of disease progression and therapy response, especially in cancer.

Remote temperature monitoring, which can benefit both the patients and the healthcare system, has been proposed^6,7^. To achieve this, reliable telemetric temperature sensors are needed. Implantable microchips that transmit temperature to an external transponder have been used for various purposes, including farm animals^8^, pets^9^, and experimental rodents^10^. However, these miniature wireless transponders are either too large to be used in tumors or subject to electromagnetic interference^10^.

In this study, battery-free chips were used to transmit reliable temperatures and are small enough to be implanted into growing tumors on small animals in order to capture both basal and tumor temperature dynamics. Three preclinical models: melanoma (B16), breast cancer (4T1) and colon cancer (MC-38), and their corresponding cancer therapy: adoptive T cell transfer, AC-T chemotherapy, and anti-PD-1 immunotherapy were used. Body and tumor temperature responses were recorded multiple times daily. Additionally, temperatures between the treatment and the control (no treatment) groups were compared.

This study provides the basis of relating the metabolic activity (represented by the temporospatial dynamics of the temperature) to tumor progression and cancer therapy. Our preclinical study suggests that high precision in vivo temperature monitoring can detect therapeutic responses by tracking tumor and body temperature changes following cancer treatment, especially immunotherapy. We postulate that changes in the temperature of the local tumor environment may be an early predictor to RECIST response for various forms of cancer therapy.

## Methods and materials

### In vivo temperature measurement

All animal usage and experimental procedure protocols were reviewed and approved by the Institutional Animal Care and Use Committee (IACUC) of the University of Minnesota. All experiments were performed in accordance with relevant guidelines and regulations. All methods are reported in accordance with ARRIVE guidelines.

All mice had 4–6 mm subcutaneous tumors placed in the right flank. Two sets of temperatures were collected: the basal temperature which reflects the body baseline and the tumor temperature which indicates the internal temperature of the tumor. The basal temperature was measured at the subcutaneous space on the left flank of the mouse using a temperature chip (2 mm diameter, 12 mm length) that transmits data to an external transponder at 400 kHz. The tumor temperature was measured by placing a temperature chip (1 mm diameter, 10 mm length) in the center of the tumor that transmits data at 134.2 kHz. Both chip types are built with two parts on both ends: the temperature sensing unit (made of thermistor and ASIC, respectively) and an RF transmitter/receiver unit. A thin (< 0.3 mm) anti-migration sheath covering the temperature-sensing end was used to help immobilize the implants. The chips used in this study are commercially available, their specification and performance are summarized in Supplementary Information S1 (1, Implantable Temperature Transponders). No hindrance of animal mobility nor sign of discomfort following the chip implantation was observed.

Chip implantation was performed under general anesthesia with Ketamine/Xylazine. Sterile chips were loaded in a trocar and the skin's surface was prepped with 70% Ethanol. During the procedure, a small incision (~ 2 mm, 5–10 mm away from the measurement side) was made to allow the chip to enter underneath the skin. The chips were then gently pushed with the temperature-sensing end facing front, below the dermis (left flank) or directly into the tumor (right flank). The incisions were then closed so that the chip stayed completely within the body.

Mice were individually caged during the temperature recording period. During the contactless measurements, animals were reached by placing the transponders below the cage without touching the cage to minimize disturbance to the animals. Information including the date, time, basal temperature, and tumor temperature was recorded. Mice were identified by both the cage number and the chip ID associated with the 134.2 kHz GTA chip.

### Cancer models

The melanoma cell line (B16-F10) was obtained from ATCC. Cells were cultured in DMEM with 10% FBS and Pen-Strep. The TNBC cell line (4T1) was obtained from ATCC. Cells were in RPMI-1640 with 10% FBS and Pen-Strep. The colon adenocarcinoma cell line (MC-38) was obtained from Kerafast, provided by James W. Hodge and Jeffrey Schlom at National Cancer Institute. Cells were cultured in DMEM with 10% FBS, 2 mM glutamine, 0.1 mM nonessential amino acids, 1 mM sodium pyruvate, 10 mM HEPES, 50 μg mL^−1^ gentamicin sulfate, and Pen-Strep.

C57BL/6J mice (female, 8–10 weeks) were obtained from Jackson Laboratory (Bar Harbor, ME). BALB/c mice (female, 8–9 weeks) were obtained from Envigo. When > 85% confluence was reached, the cells were detached by 0.05% Trypsin-EDTA and resuspended in phosphate-buffered saline in 20 million cells mL^−1^. Tumors were inoculated by injecting 50 μL of cell suspension subcutaneously into the hindlimb of mice. Experiments were performed 9–13 days after tumor seeding when a tumor diameter of 4–6 mm was obtained. Animals were randomized into the control group and the treatment group.

### Cancer therapies

Adoptive T cells transfer for melanoma was achieved through intravenous (IV) transfer of TRP-2 T cells, which are CD8 + T cells bearing a genetically encoded high-affinity receptor against the TRP-2 tumor antigen from B16 tumor cells^11,12^. TRP-2 specific T cells (at least 95% CD8 + Thy1.1 +) were isolated using a CD8 + T cell isolation kit. The TRP-2 T cells were further stimulated in vitro with IL-12 (2.5 ng/ml) and IL-2 (200U/ml) following protocols described by Tucker et al.^13^. 1 million activated TRP-2 T cells were transferred via IV to recipient mice through retro-orbital injection under general anesthesia with Isoflurane.

AC-T chemotherapy, which has been used clinically for treating breast cancer^14^, for 4T1 tumor (TNBC model) was achieved by intratumoral injection of chemotherapeutic agents (0.1 ml) made up of Doxorubicin Hydrochloride (2 mg/kg), Cyclophosphamide (50 mg/kg), and Paclitaxel (5 mg/kg) solution in saline.

Anti-PD-1 immunotherapy, which has been approved for treating MSI-H and dMMR colorectal cancer, has also been demonstrated to be effective in preclinical colon cancer models including MC-38^15,16^. Immunotherapy was delivered by intraperitoneal injecting 100 μg of antibody (InVivoMAb anti-mouse PD-1, Clone RMP1-14 from Bio X Cell) on days 1, 3, and 5 on the mice bearing MC-38 tumor.

### Study design

**Figure 1:**
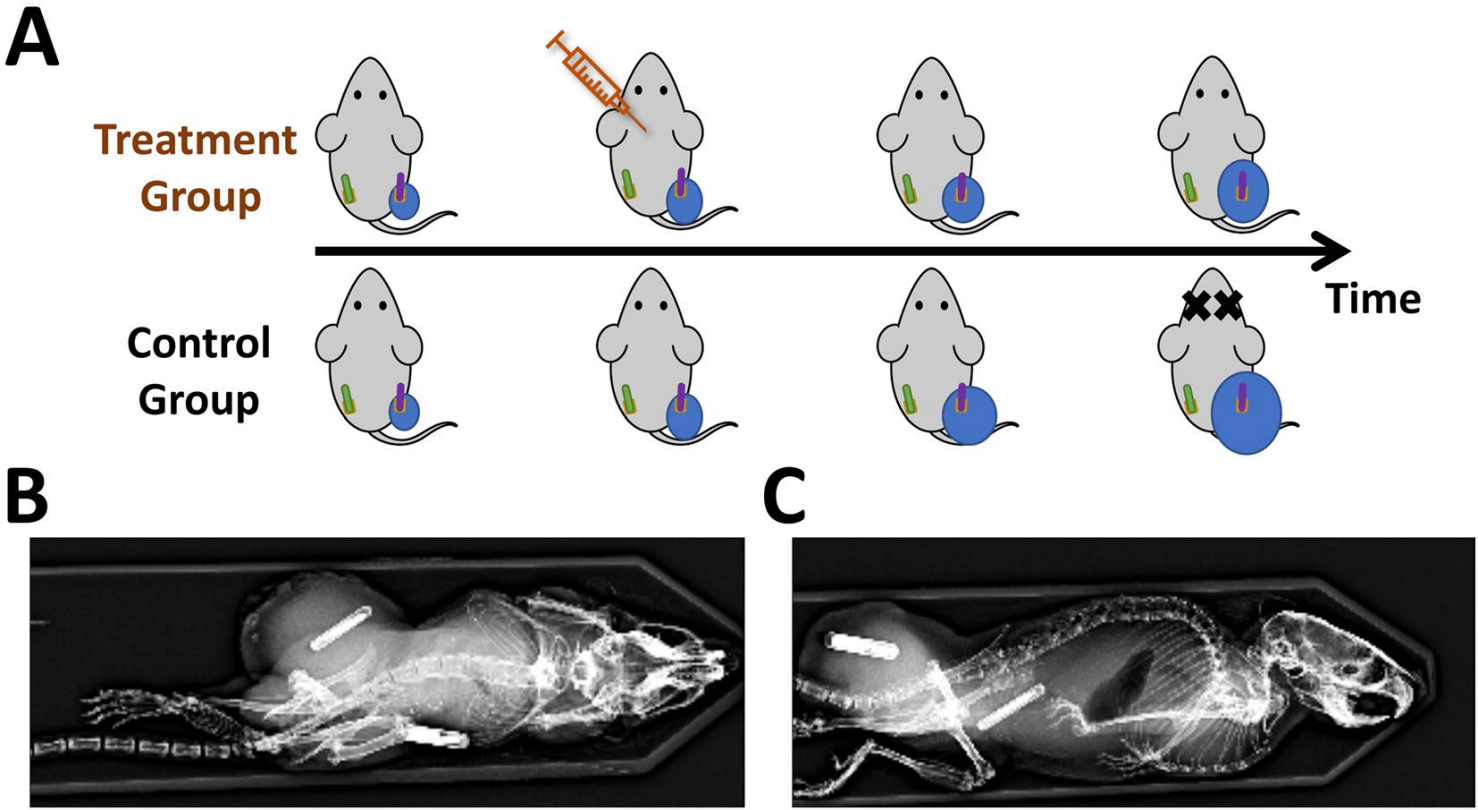
Study design and temperature chip placement verified by X-ray. (**A**) For each cancer model and its corresponding therapy, mice were divided into the treatment group and the control group. Both groups followed the same recording protocol. (**B**) Post-mortem X-ray image of a B16 tumor on a C57BL/6J mouse. (**C**) Post-mortem X-ray image of a 4T1 tumor on a BALB/c mouse. Note that two chips were implanted on both tumor-bearing mice: one on the left flank the other one the right flank inside the tumor. Magnification X-rays were acquired with Faxitron Specimen Radiography System (Hologic, Santa Clara, CA).

For each cancer model, a cohort of tumor-bearing mice following inoculation was divided into two groups: the treatment group and the control group. Cancer treatment was given to the treatment group only, as illustrated in Fig. 1A. Examples of experimental mice implanted with a pair of temperature chips for simultaneous recording of body temperature and tumor temperature are shown in Fig. 1B,C.

Mice were euthanized when the tumor reached endpoints (16 mm length or 2 cm^3^) or showed other signs of sickness or study-related complications, including skin ulceration. Data from the mice whose chip was found either dislodged from the tumor or not fully embedded under the skin were excluded from further analysis. The number of mice included in each group was no less than 6.

### Data processing and statistics

Within each cancer model, for each mouse, two temperatures readings were recorded at time t_0_: T_tumor_(t_0_) and T_body_(t_0_), and the difference in temperature between the tumor and the body is given by ∆T(t_0_) = T_tumor_(t_0_)- T_body_(t_0_). For comparison between two groups, ∆T_A_(t) or ∆T_B_ (t) describes the average of ∆T within group A or group B. “t” is given in days, when t = 0 represents the date of tumor inoculation (start of the tumor). Data are presented with average ± standard deviation.

Welch's t-test, using the two-tailed distribution and unequal variance, compares between two groups on the same day. P values were calculated to evaluate the significance of the difference between the two groups. P < 0.05 is considered to be statistically significant.

## Results

### Temperature response of melanoma treated with adoptive T cell transfer immunotherapy

**Figure 2:**
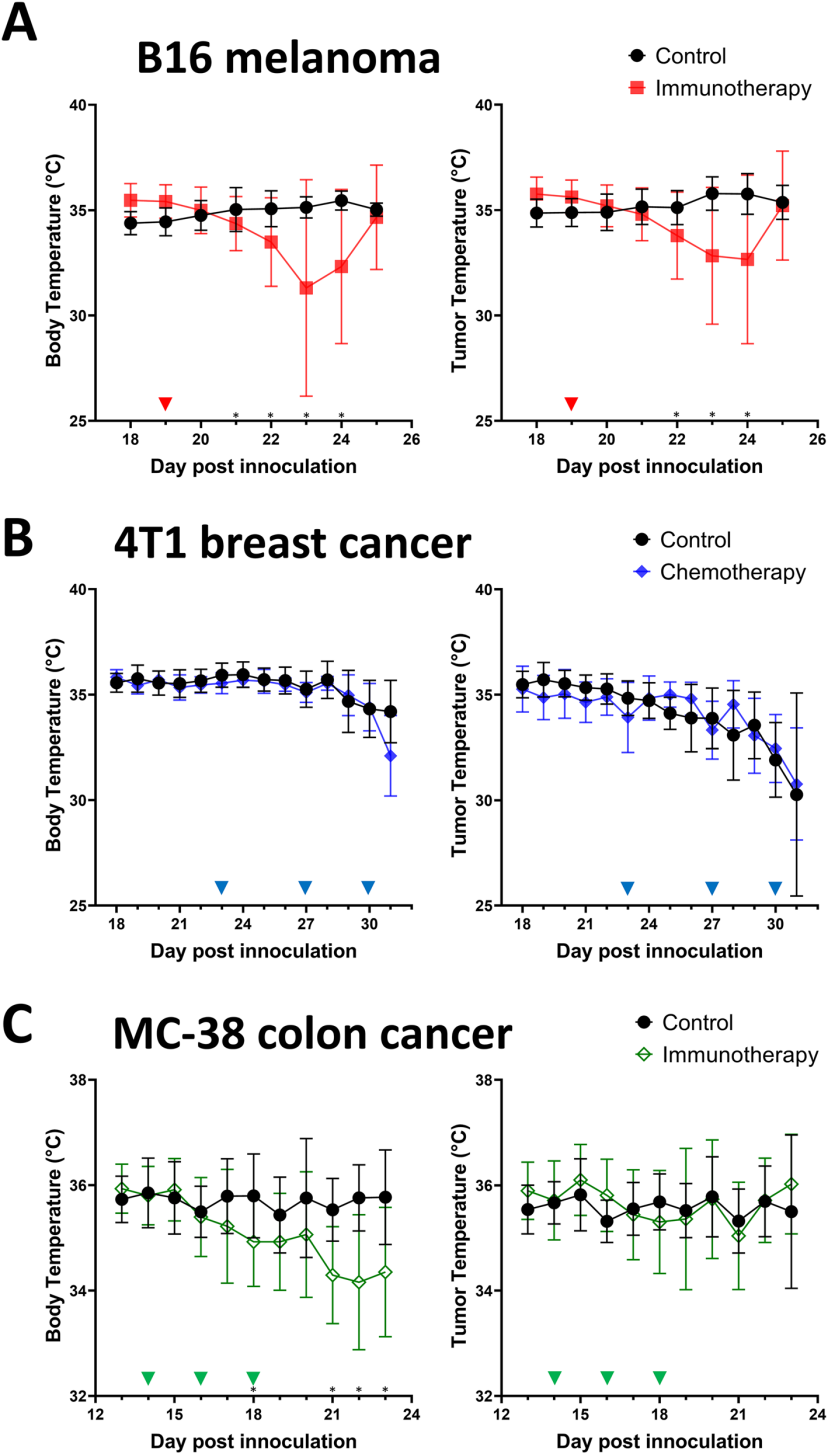
Temperature of the body and the tumor, in each of the cancer model, responding to corresponding cancer treatment. (**A**) B16 melanoma-bearing mice (control, n = 10) and its response to TRP-2 T cells adoptive transfer (immunotherapy, n = 8). (**B**) 4T1 TNBC-bearing mice (control, n = 10) and its response to AC-T chemotherapy (n = 6). (**C**) MC-38 colon cancer-bearing mice (control, n = 11) and its response of receiving anti-PD-1 antibody (immunotherapy, n = 7). Data are presented with average (symbol) ± standard deviation (error bar). Left, body temperature; right, tumor temperature. Days when therapy was administered are labeled with ▼. Days with statistically significant difference (P < 0.05) between the control and the treatment group is denoted with *.

The B16-tumor-bearing mice had stable body and tumor temperature. The temperature chips were placed between day 14 and day 17 after the B16 tumor inoculation. In the control group, both the body and tumor temperature remained relatively steady over the recording period with daily temperature readings of 34.4 ± 0.7 to 35.4 ± 0.5 °C and 34.9 ± 0.7 to 35.8 ± 0.8 °C, respectively, as shown in Fig. 2A between days 18 and 25, during which the tumor growth was unhindered before the animals have to be euthanized.

The temperature response to adoptive T cell transfer was conspicuous with a decrease of both the body and the tumor temperature, following the immunotherapy on day 19, as shown in Fig. 2A. Body temperature started decreasing immediately following the immunotherapy, reaching the minimum on four days after the immunotherapy (day 23): 31.3 ± 5.1 °C compared to 35.5 ± 0.8 °C prior to the treatment. The body temperature difference of 2 groups (control vs immunotherapy) between days 21 and 24 was statistically significant (p = 0.022, 0.004, 0.004 and 0.004). The tumor temperature also decreased after immunotherapy, with reading nadir on day 24 at 32.7 ± 4.0 °C compared to 35.8 ± 0.8 °C on day 18 before the therapy. The tumor temperature difference of the 2 groups (control vs immunotherapy) between days 22 and 24 was statistically significant (p = 0.011, 0.002 and 0.009). Interestingly, for both measurements, the gap between the treatment and the control diminished on day 25.

### Temperature response of TNBC being treated with chemotherapy

In the 4T1 TNBC model, chips were implanted on day 18 following the inoculation. In the control group, the body temperature was stable till day 28, temperature ranging between 35.2 ± 0.8 and 35.9 ± 0.6 °C, before starting to decrease to 34.2 ± 1.5 by day 31, when the tumor burden started to take its toll, as shown in Fig. 2B. A steady decline of the tumor temperature was noticeable, from 35.5 ± 0.6 to 32.1 ± 1.9 °C towards the end of recording, as shown in Fig. 2B.

AC-T chemotherapy was given on days 23, 27, and 30. Chemotherapy caused no statistical differences (chemotherapy vs control) in either body or tumor temperature within the course of recording, as shown in Fig. 2B. Similar to that of the control group, the body temperature decreased between days 29 and 31, from 33.5 ± 1.6 to 30.6 ± 4.8 °C. Chemotherapy did not reverse the downward trend of tumor temperature decline, even though the average tumor temperature briefly increased after the 1st and the 2nd dose of chemotherapy by 0.9–1.5 °C on days 23 and 27; however, the difference (chemotherapy vs control) was not statistically significant.

### Temperature response of colon cancer being treated with anti-PD-1 immunotherapy

The body and tumor temperature of the MC-38 colon cancer model was relatively unchanged throughout the course of tumor growth. Chips were placed between days 11 and 14. In the control group, the body temperature stayed between 35.4 ± 0.7 and 35.9 ± 0.7 °C, while the tumor temperature fell between 35.3 ± 0.4 and 35.8 ± 0.7 °C, as shown in Fig. 2C.

Anti-PD-1 immunotherapy caused a significant reduction in body temperature but did not seem to affect tumor temperature. The first dose of antibodies was given on day 14 and the course of immunotherapy lasted till day 18. The body temperature decreased from 35.9 ± 0.5 °C prior to treatment to 34.2 ± 1.3 °C on day 22, as shown in Fig. 2C. The difference (control vs immunotherapy) was statistically significant on day 18 (p = 0.026) and between days 21 and 23 (p = 0.006, 0.027 and 0.035). The tumor temperature remained relatively unaffected, staying between 35.3 ± 1.0 and 36.0 ± 0.9 °C, without a statistically significant difference from the control group, as shown in Fig. 2C.

### Temperature difference between the tumor and the body

**Figure 3:**
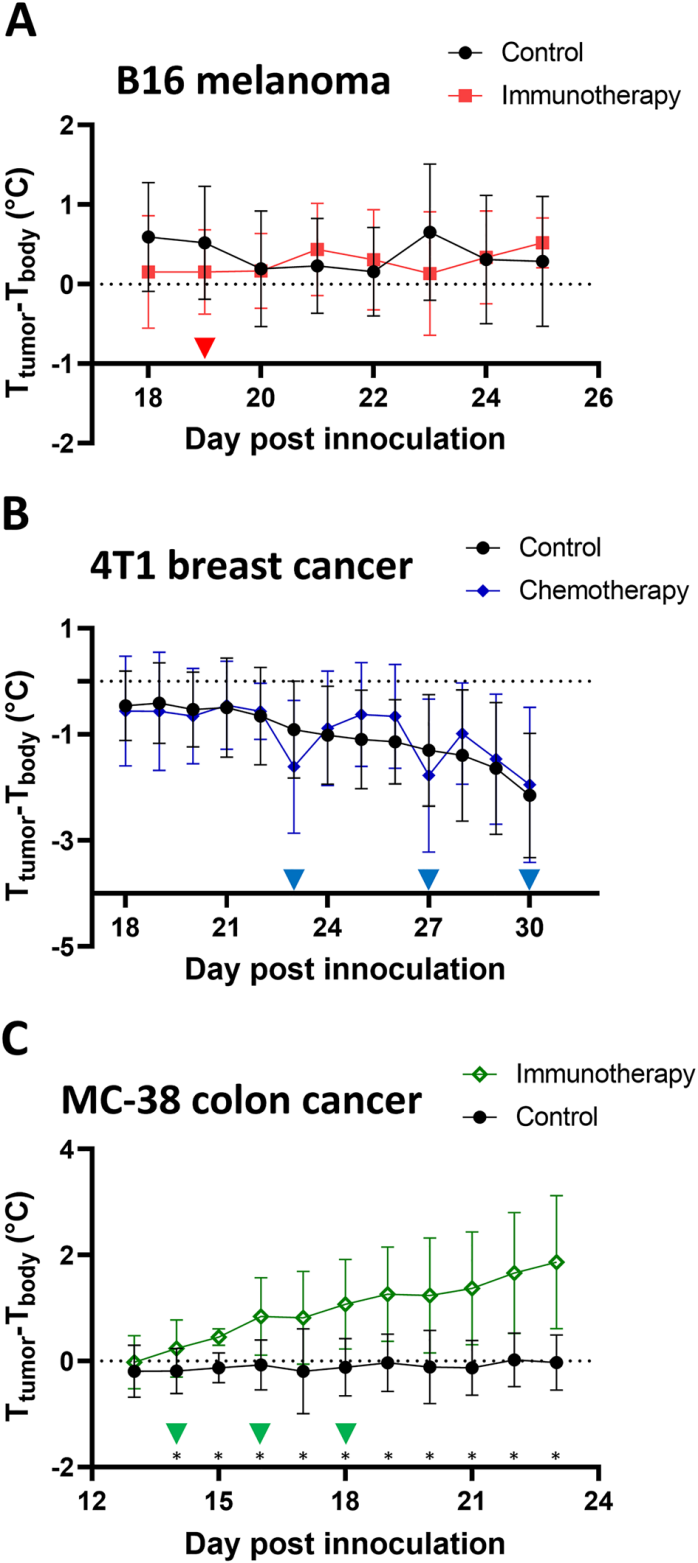
Temperature difference (∆T, tumor temperature minus body temperature) between the tumor and the body, and their response to cancer treatment. (**A**) B16 melanoma adaptive T cell transfer immunotherapy. (**B**) 4T1 TNBC AC-T chemotherapy. (**C**) MC-38 colon cancer anti-PD-1 immunotherapy. Data are presented with average (symbol) ± standard deviation (error bar). Days when therapy was administered are labeled with ▼. Days with statistically significant difference (P < 0.05) between the control and the treatment group is denoted with *.

As shown in Fig. 3A, B16 tumors consistently presented a higher temperature than the basal body temperature in this paired comparison. Despite a change in both body and tumor temperatures in response to the adaptive T-cell transfer, the gap between the two temperatures remained largely unchanged throughout the course of the tumor development and cancer treatment. In the control group, the tumor was 0.16 ± 0.6 °C to 0.65 ± 0.9 °C warmer than the body; while in the treatment group, the tumor remained 0.13 ± 0.8 °C to 0.52 ± 0.3 °C warmer than the body. The ∆T (tumor temperature minus body temperature) between the two groups was not statistically significant.

The evolution of ∆T in the 4T1 model is presented in Fig. 3B. The 4T1 tumor was cooler than the body, with a vast majority of recorded ∆Ts being negative. In the control group, the ∆T was wider with the tumor progression: the ∆T decreased from − 0.46 ± 0.6 to − 2.15 ± 1.2 °C during the course of temperature monitoring. In the AC-T chemotherapy group, the descending trend of ∆T remained unchanged, corresponding to a drop of ∆T from − 0.56 ± 1.0 °C to − 1.95 ± 1.4 °C. AC-T tends to slow the decrease of ∆T by a few days, as seen by an elevation of ∆T in the chemotherapy group compared to that of the control group on days 24–26 and 28–29, corresponding to 1–3 days after the 1st and 2nd dose of chemotherapy. The dips of ∆T on days 23 and 27 aligned with the IT administration of AC-T chemotherapy. Despite the variation of ∆T following chemotherapy, the differences were not statistically significant compared to those in the control group.

The distinctive feature of the ∆T in the MC-38 model with and without the anti-PD-1 immunotherapy is presented in Fig. 3C. The MC-38 tumor has a similar temperature to the body temperature yet slightly cooler than the body without any treatment. The ∆T remained mostly unchanged throughout the tumor progression, staying between − 0.19 ± 0.5 °C and 0.02 ± 0.5 °C. However, the ∆T is pronounced following the anti-PD-1 immunotherapy. In this group, the paired ∆T gradually increased from − 0.02 ± 0.5 to 1.86 ± 1.3 °C. The difference (control vs immunotherapy) was statistically significant between day 14 and day 23 (p = 0.025, < 0.001, 0.009, 0.014, 0.005, 0.004, 0.009, 0.005, 0.016 and 0.013). Given that the tumor temperature remained stable with or without the immunotherapy, the gap between the two temperatures was largely attributed to the decrease in body temperature in response to anti-PD-1 immunotherapy.

## Discussions

### Temperature sensing and therapeutic response

The goal of this study is to provide basal and tumor temperature measurements in cancerous models that showed disease progression (control group) compared to those (treatment group) receiving their corresponding regimens (e.g., immunotherapy or chemotherapy) which are known to result in objective response in each of the models. We hypothesize that changes to in vivo temperature measurements of both the tumor bed and the basal body temperature can allow us to identify an early response to cancer therapies.

In this study, we found that tumor temperature can be similar to that of the body (such as MC-38), higher than body temperature (such as B16) or lower than the body temperature (such as 4T1). The ∆T can stay relatively unchanged during tumor progression (such as B16 and MC-38), or become wider (such as 4T1, from an average of 0.56–1.95 °C) before the animals were overcame by the tumor burden.

Statistically difference in temperature can be observed as early as 1–2 days following the onset of immunotherapy in our preclinical models. In the B16 model, the earliest significant differences between the treatment and the control group were observed to be 2 days (body temperature) and 3 days (tumor temperature) respectively. In the MC-38 model, a significant difference in ∆T was observed only 1 day following the first dose of anti-PD-1 antibody, a difference in body temperature when compared to the control groups was first observed on day 4 after the initiation of anti-PD-1 immunotherapy. For comparison, tumor growth differences are either too small to differentiate or take a much longer time to show difference between groups, in the same model receiving the same treatment regimen. For instance, in the B16 model, “TRP-2-specific T cells infiltrate the tumor but do not affect tumor growth”^11^. In the MC-38 model, the difference in tumor growth is only observable after 13 days of anti-PD-1 immunotherapy^13^.

Cancer angiogenesis and deregulated cellular energetics are hallmarks of cancer. “Thermal profiling” offers an additional dimension of cancer characteristics not available by conventional imaging and biopsy. The simple, low-cost, and non-invasive telemetric temperature sensing for monitoring cancer progression and treatment response can be a viable addition to existing tools for cancer diagnosis. Nevertheless, tumor response is likely multifactorial, and the level of response is on a continuum. More in-depth research with the temperature transponder joining the arsenal of tools to advance therapeutic efficacies is warranted, given this proof-of-concept study.

### Clinical significance

Early detection of tumor response to therapeutic intervention has been a long-standing goal for physicians. Existing procedures of screening are either prohibitively resource-intensive and expensive or are unable to provide direct quantitative estimates of the relevant physiological parameters for accurate classification. As shown in this study and discussed previously, the measurable and statistically difference in temperature can precede the difference in tumor sizes in response to cancer immunotherapy, suggesting that the temperature response can be an early indicator of treatment response, without involving complex imaging or blood testing. Knowing the drug response to a specific tumor is clinically significant for several factors. For example, the pseudoprogression of immunotherapy is common when the tumors grow larger following the therapy due to large amounts of tumor-infiltrating cells before eventually shrinking^17^. Being able to distinguish between an actual disease progression (not responding to immunotherapy) from pseudoprogression can help physicians make an informed decision on cancer management within a much shorter time frame, as compared to waiting for months by tracking changes in tumor volume. In addition, it typically takes a much longer time to determine the response of a solid tumor to immunotherapy (described in iRECIST^18^) than conventional approaches, such as chemotherapy, surgery, or radiation therapy. However, from our study, the temperature responses can take place within a few days before changes in the size of the tumor. Being able to differentiate tumor response earlier can be both cost- and life-saving. Our proposed “telemetric thermal profiling” strategy could offer an inexpensive option to supplement established practice, therefore enabling physicians to make quicker and more informed decisions in cancer care.

One of the unique features of the temperature chips is that they can be FDA-approved to be permanently implanted into the tumor, as demonstrated previously^19,20^. After implantation, this technology enables on-demand monitoring of the tumor temperature that can be carried out at any time and on any frequency basis. This flexibility is helpful in a resource-limited setting, such as from the patent’s home. The recorded temperature can be transmitted wirelessly to an external transponder without hindering the movement of the subject. This telemetric temperature sensing technique could facilitate the development of a cloud-based patient monitor system, therefore mitigating the laborious need for patients to present in person at a healthcare facility for tests and imaging.

The tumor temperature deviation from the body sheds light on the physiological features of the tumors. The tumor can have a temperature higher than body temperature, which serves as the basis of infrared (IR) thermography for breast cancer screening^21^ and has been demonstrated for a handful of cancer types^22^. However, the tumors can have a lower temperature than surrounding tissues in various preclinical models^23,24^ and clinical observations^25^ with Infrared thermography. In this study, the tumor temperature can be higher than body temperature, as shown with the B16 tumors, or lower than body temperature, as shown with MC-38. Moreover, the tumor progression changes the energy balance, therefore causing the tumor to shift from “hotter” to “colder” than the body, as shown in our 4T1 tumor model. The trend of tumors getting “colder” with the increase in volume is also noted in some preclinical models^23,24^.

Non-invasive monitoring of body and tissue temperature is especially important in thermal medicine. Thermal therapy, the manipulation of body or tissue temperature, has a broad medical application including cancer. For example, depending on the temperature and exposure time, heating can lead to direct cell death or activate vascular, metabolic, and immunologic parameters of the tumor microenvironment, which may play an additional role in radiochemosensitization^26^. Thermometry sensors are important for an accurate evaluation of the quality of hyperthermia treatment and the calculation of the thermal dose delivered. Moreover, advanced heating systems demand extensive thermometry for the effective utilization of temperature feedback power control^26^.

### Potential mechanism of temperature changes

The local temperature distribution and the energy balance within the tissue is described by the Pennes bioheat transfer equation^27^

1 $$\rho {c}_{p}(T)\frac{\partial T}{\partial t}-\nabla \left(k\left(T\right) \nabla T\right)+{w}_{b}{c}_{b}\left(T-{T}_{a}\right)={Q}_{met}$$

where T and t are temperature and time; ρ, c_p_ and k are density, specific heat, and thermal conductivity of the biological tissues; ρ_b_, c_b_, w_b_, T_a_ and Q_met_ represent blood density, specific heat, perfusion rate, arterial temperature, and metabolic heat generation, respectively.

Between tumors and normal tissues, the distinctive difference in the metabolic heat generation and blood perfusion are the major factors affecting the bioheat transfer, while other thermal properties (density, specific heat, and thermal conductivity) are very similar to those of normal tissues (less than 10%, except for fat). Metabolic heat generation (Q_met_) of tumors can be 2.5 ×^28^ to 60 ×^29^ of that of normal tissue. Blood perfusion (wb) varies significantly depending on the tumor type and pathological conditions; it can increase with angiogenic increases in vascularity, leading to as much as 50 × than that of normal tissue^29^. However, necrotic tumors tend to have less blood perfusion due to tumor growth outpacing blood supply^30^.

The pathophysiology of the tumor, either by tumor progression or external intervention, can also affect the tumor’s thermal properties. For instance, it has been reported that the metabolic heat production (Q_met_) of a tumor is inversely proportional to the doubling volume time^31^. In response to cancer therapy, there is higher heat generation (fourfold) by mitochondria during apoptosis compared to resting states^32,33^, as well as a positive correlation between oxygen saturation and mitochondrial heating rate^34,35^. The reduction of tumor temperature has been associated with necrosis (therefore reduced metabolism) accompanied by vascular disruption^36^. Taken together, tumor temperature changes, both spatial and temporal, have significant diagnostic value by reflecting tumor physiology and response to treatments.

It is also worth noting that body temperature changes with tumor progression and immunotherapy, as shown in Fig. 2, even though the housing temperature remains consistent. Body temperature changes can be reflected in T_a_. Other studies have found that the tumor-bearing mice “feel colder” than non-tumor-bearing mice, and this is not well understood^37^. However, the relation between metabolic stress associated with tumor growth and thermoregulation remains unclear. Moreover, mechanistic pathways linking metabolic cold stress and antitumor immunity are not yet defined. It has been suggested that immunological defenses against tumors are energetically costly, therefore leading to the activation of thermogenesis^38^.

In this study, the actual mechanism contributing to temperature difference and its changes remains to be uncovered. Based on our observation, tumor growth is accompanied with formation of necrosis core and angiogenesis at the outer edge of the tumors. B16 is the most necrotic among 3 models. The B16 tumors are soft and fluid, while 4T1 tumors tend to be more solid and stiff. The 4T1 tumors present “pale” inner parts compared to “pink” edges, suggesting a lack of blood supplies to the center of the tumor. MC-38 is considered as a “hot tumor”, characterized by high tumor mutational burden, increased expression of PD-L1 and IFN-γ signaling, and high T-cell infiltration, in contrast to “cold” tumor like B16.

In this study, no differences between tumor and body, or between control/treatment groups was observed in the chemotherapy-treated TNBC model. While cancer immunotherapy typically relies on tumor-filtrating lymphocytes (TILs) to be effective, chemotherapy drugs act on cancer cells directly. AC-T chemotherapy was administered intratumorally (other than IV) to increase the localized cytotoxic effect without increasing systemic toxicity. The lack of noticeable response for the AC-T regimen remained to be investigated.

### Limitations

The temperature measurements for this study were subject to variation due to some factors, including animal activity, circadian rhythm, and variation among individuals. The uncertainty of measurements is affected by the accuracy and repeatability of the temperature chips, in addition to the inhomogeneity of temperature within the tumor. The variation and uncertainty of temperature measurements are quantitatively discussed in Supplementary Information S1 (2. Variation in vivo temperature and 3. Uncertainty of temperature measurements). This study uses means between groups and observe deviation, such deviation can diminish for individual subjects.

An important question is whether tracking the temperature of an individual is sufficient to differentiate a responder from a non-responder to immunotherapy. A few aspects need to be addressed and rigorously evaluated: (1) optimal time window and threshold to differentiate responder from non-responder remain to be investigated. (2) reliable data processing approach to distinguish temperature change due to tumor progression/response from the concurring and periodic circadian pattern (~ 1 °C of variation) remains to be studied. The day-night patterns of core temperature tend to be consistent, as shown by time-course of core temperature measured by radiotelemetry for days.^39^ (3) subject-specific baseline temperatures need to be established. Even though the temperature difference among a cohort of mice is substantial (1–3 °C), the temperature fluctuation is usually much smaller (< 1 °C) at individual level (without tumor or therapy). However, this an individual-level baseline is subject to perturbation from a wide range of environmental factors (up to 2 °C). (4) Algorithms or machine learning (ML)-based classification for precise quantitative estimation from the unwarranted noise are necessary to ensure its performance. (5) Artificial intelligence (AI)-empowered automation can improve the efficacy of the method in differentiating suspected non-responders at various cancerous stages. (6) Sensitivity and specificity of different classification strategies are to be investigated and quantified.

The relation between temperature and cancer pathology has not been investigated in this study. For example, the 4T1 tumor is known to be able to spontaneously metastasize from the primary tumor to multiple distant sites^40^, similar to clinical breast cancer. However, the correlation between the severity of metastasis and the temperature response was not investigated in this study. In-depth analysis will require careful pathological examination of the tumor microenvironment. For example, the population and composition of lymphocyte infiltration, the extent of cell death (both necrosis and apoptosis), and changes in tumor vascularization at different time points following the cancer therapy.

### Opportunities and future directions

While this study is a proof-of-concept, there are a number of hardware improvements this telemetric system can make, including accuracy, size, multiplexing capability, and automation, as discussed in Supplementary Information S1 (4. Potential hardware improvements).

While this study relies on implantable syngeneic tumor models, especially the B16 and the MC-38 which are known to be responding to the corresponding immunotherapy, more cancer models (such as transgenic cancer-prone mice, metastases and carcinogen induced tumors) and various forms of immunotherapies (including those not responding) must be evaluated to fully appreciate the underlying mechanism of temperature response.

In addition, the GTA 134.2 kHz is a platform ASIC that can be integrated with other sensor types (pH, pO_2_, and glucose). It is well known that the tumor microenvironment is characterized by acidic pH, oxygen depletion, or glucose depletion compared to normal tissues^41^, however, how these analytes change with tumor growth and/or response to cancer treatment is yet to be uncovered. The chip that can be permanently implanted and the telemetric sensing technique we presented here can open up future discoveries.

### Conclusion

In summary, this study provides the basis for monitoring temperature during tumor progression and therapeutic response to chemotherapy and immunotherapies. Our preclinical study suggests that high precision in vivo temperature monitoring can detect therapeutic responses to treatments by following tumor temperature changes during the treatment therapeutic window. Tracking of in vivo thermal activity was realized with the precision and accuracy of the implanted devices, which could offer earlier treatment assessment to patients without requiring complex imaging or lab testing.

On-demand monitoring of tumor temperature could be used for confirming treatment efficacy or regiment course adjustment. Therefore, tumor telemetric temperature sensing has the potential to facilitate a more efficient management plan and a reduction of patent burden. We posit that the inexpensive, accurate and telemetric temperature sensing has the promise of an accurate in situ screening and diagnostic approach for cancer management. Furthermore, the fundamental scientific premise of the present technique holds the potential of opening new vistas in rapid and affordable digital healthcare for early detection of tumor response.

## Supplementary Information

Supplementary Information.

## Data Availability

The datasets generated during and/or analyzed during the current study are available from the corresponding author on reasonable request.

## Abbreviations

AC-T: Doxorubicin hydrochloride (Adriamycin) and cyclophosphamide, followed by paclitaxel (Taxol)
ARRIVE: Animal research: reporting of in vivo experiments
ASIC: Application-specific integrated circuit
ATCC: American type culture collection
Anti-PD-1: Programmed cell death protein 1 (PD-1) inhibitor
DMEM: Dulbecco's Modified Eagle Medium
dMMR: Mismatch repair deficient
EDTA: Ethylenediaminetetraacetic acid
FBS: Fetal bovine serum
GTA: Geissler Temperature ASIC™
HEPES: 4-(2-Hydroxyethyl)-1-piperazineethanesulfonic acid
MR: Magnetic resonance
MSI-H: Microsatellite instability-high
IACUC: The institutional animal care and use committee
iRECIST: Modified response evaluation criteria in solid tumors in cancer immunotherapy trials
IV: Intravenous
Pen-Strep: Penicillin G and streptomycin
RECIST: Response evaluation criteria in solid tumors
TcR: T-cell receptor
TNBC: Triple-negative breast cancer
TRP-2: Tyrosinase related protein-2
