# Supplementary material for: Tumor therapeutic response monitored by telemetric temperature sensing, a preclinical study on immunotherapy and chemotherapy
Source: Sci Rep. 2023 May 12;13:7727. doi: 10.1038/s41598-023-34919-w (PMC10182083; doi:10.1038/s41598-023-34919-w)
Supplement: Supplementary file 1 — Supplementary Information. [file 41598_2023_34919_MOESM1_ESM.docx]

**Supplementary Information**

Tumor therapeutic response monitored by telemetric temperature sensing, a preclinical study on immunotherapy and chemotherapy

Contents

[1. Implantable Temperature-sensing Transponders 1](#_Toc130481397)

[2. Variation *in vivo* temperature 1](#_Toc130481398)

[3. Uncertainty of temperature measurements 2](#_Toc130481399)

[4. Potential hardware improvements 2](#_Toc130481400)

[5. Statistical approaches 2](#_Toc130481401)

[Reference for Supplemental Information 2](#_Toc130481402)

## 1. Implantable Temperature-sensing Transponders

The body temperature is measured by IPTT-300 implantable RFID microchip made by BMDS (Bio Medic Data Systems parts of Avidity Science) [1]. The commercially-available system has been used in a variety of *in vivo* applications [2-4]. Transponders are encased in glass, preloaded in a disposable needle assembly and Injected with a syringe-like action. A DAS-8007 wireless reader (BMDS) was used to retrieve the temperature from corresponding transponders [5].

The tumor temperature is measured by Geissler Temperature ASIC (GTA) transponders, provided by Geissler Corporation (Plymouth, MN). The glass encapsulated microchip has a built-in temperature biosensor and a Radio-Frequency Identification (RFID) tag that offers a unique and unalterable identification. A proprietary reader (provided by Geissler Corporation) was used to retrieve chip ID and temperature from corresponding GTA transponders.

According to the specifications provided by BMDS, IPTT-300 chip has a temperature range from 20.0 °C to 42.0 °C, a resolution of 0.1 °C, an accuracy of 0.2 °C (between 34 °C to 42 °C) or 0.5 °C (between 30°C and 34°C). An independent study shows that the GTA chips has a temperature range no smaller than the physiologically relevant temperature range (34-43 ˚C), a resolution of 0.112 °C, an accuracy of better than 0.1 °C.

## 2. Variation *in vivo* temperature

The temperature measurements for this study were subject to variation and uncertainty due to some factors. The most noticeable being animal activity: the authors have noted that handling and restraining a mouse can cause a temperature change as much as 2 ˚C largely due to stress and anxiety physiological responses. Therefore, a few strategies have been implemented to minimize the disturbance to the animals during temperature measurement, including placing the wireless transponder recording temperature below the cage without physically touching the cage and the animals, gently moving the cages when necessary, and keeping the vivarium quiet during recording. Temperature variation (as much as 1 ˚C) at different times of the day (circadian rhythms) was also noted. Therefore, multiple temperatures were taken at different times of the day (typically morning, afternoon, and night) to track the temperature changes effectively. Temperature variations among individuals (1-3 ˚C) are common, in addition to the menstrual cycle of the female subjects, therefore a large number of mice is needed for each group to ensure revealing the trends of temperature changes from tumor growth and therapeutic response.

## 3. Uncertainty of temperature measurements

The miniaturized chips, which convert the temperature to an electric signal, digitize and transmit to external transponders, inevitably introduce uncertainty in measurement during the process. Moreover, temperature within given tumors is not uniform. One study using thermal imaging showed that between the hotter and cooler regions can vary by a maximum temperature difference of 1- 5 ˚C, and this gap widens with tumor growth [6]. Therefore, the actual location of the temperature-sensing element of the chip and its contact area within the tumor may have an impact on the readouts.

## 4. Potential hardware improvements

While this study is a proof-of-concept, there are a number of hardware improvements the telemetric can make. First and foremost, there is room for improvement of the chip's design: the temperature accuracy, size, and multiplexing capability. Currently, only one mouse is housed in one cage as the RFID reader of chips operating at the same frequency is not able to distinguish which chip is being read when multiple chips are within the transmission range. Within one subject, monitoring more locations with thermometry sensors offers a more complete knowledge of the temperature field, which is desirable for tumor thermal therapy. In this study, the readers for the data recording and transfer are still done manually, which will require lab staff working at the vivarium. A reader with an automated system that records the data and stores it to a central repository will further minimize disturbance to the animals. No presence of lab staff would allow for consistent interval temperature measurements, which could reveal other trends of temperature, such as fast changes to therapy (within hours) that were impossible with laborious manners.

## 5. Statistical approaches

This study focuses on comparing observed temperature changes between two groups (treatment vs. control) to reveal associations between cancer therapy and the temperature response. T-tests were used to statistically differentiate the trends of temperature in the two groups. However, the T-test assumes a normal distribution of the data, which may not hold true in some extreme cases when a few readings were exceptionally deviated from the normal. Within such group, the likelihood of a reading lower than the average temperature is not equal to the likelihood of a reading above average. Other statistical methods, such as categorical and parametric models (e.g., Mann–Whitney–Wilcoxon test), may depict the difference in temperature response better.

## Reference for Supplemental Information

1. <https://www.avidityscience.com/control-monitoring/bmds/transponder>

2. Kokolus, K. M., Capitano, M. L., Lee, C. T., Eng, J. W. L., Waight, J. D., Hylander, B. L., ... & Repasky, E. A. (2013). Baseline tumor growth and immune control in laboratory mice are significantly influenced by subthermoneutral housing temperature. Proceedings of the National Academy of Sciences, 110(50), 20176-20181.

3. Edwards, M. M., Nguyen, H. K., Herbertson, A. J., Dodson, A. D., Wietecha, T., Wolden-Hanson, T., ... & Blevins, J. E. (2021). Chronic hindbrain administration of oxytocin elicits weight loss in male diet-induced obese mice. American Journal of Physiology-Regulatory, Integrative and Comparative Physiology, 320(4), R471-R487.

4. Langer, F., & Fietz, J. (2014). Ways to measure body temperature in the field. Journal of Thermal Biology, 42, 46-51.

5. <https://www.avidityscience.com/control-monitoring/bmds/readers>

6. Tepper, M., Shoval, A., Hoffer, O., Confino, H., Schmidt, M., Kelson, I., ... & Gannot, I. (2013). Thermographic investigation of tumor size, and its correlation to tumor relative temperature, in mice with transplantable solid breast carcinoma. *Journal of biomedical optics*, *18*(11), 111410.
